# Work productivity and activity in patients with SAPHO syndrome: a cross-sectional observational study

**DOI:** 10.1186/s13023-022-02523-2

**Published:** 2022-10-21

**Authors:** Chen Li, Heng Xu, Liang Gong, Afang Wang, Xia Dong, Kai Yuan, Guangrui Huang, Shufeng Wei, Luying Sun

**Affiliations:** 1 Department of Rheumatology, Fangshan Hospital, Beijing University of Chinese Medicine, Beijing, China; 2 Department of Traditional Chinese Medicine, Peking Union Medical College, Peking Union Medical College Hospital, Chinese Academy of Medical Sciences, No.1 Shuaifuyuan, 100730 Beijing, China; 3 School of Life Sciences, Beijing University of Chinese Medicine, Beijing, China; 4 Department of Nephrology, Fangshan Hospital Beijing University of Chinese Medicine, Beijing, China

**Keywords:** SAPHO syndrome, Work productivity, Activity impairment

## Abstract

**Background:**

Our understanding of work productivity impairment among patients with synovitis, acne, pustulosis, hyperostosis, and osteitis (SAPHO) syndrome is limited. The purpose of this study was to provide an overview of work productivity loss in SAPHO syndrome patients through the use of the work productivity and activity impairment (WPAI) questionnaire, as well as to investigate the relationship between the WPAI and other disease-related indicators.

**Methods:**

Patients for this cross-sectional study were recruited from Peking Union Medical College Hospital (Beijing, China). The questionnaires incorporating the WPAI were administered, along with the inclusion of demographic data, disease-specific measures, and general health variables. The construct validity of the WPAI was evaluated via the correlations between WPAI outcomes and other measures. Wilcoxon rank-sum tests and nonparametric Kruskal‒Wallis tests were used for the comparison of the WPAI outcomes between known groups.

**Results:**

A total of 376 patients were included, and 201 patients (53.5%) were employed. The medians (interquartile range [IQR]) of absenteeism, presenteeism, work productivity loss, and activity impairment were 0% (0–13%), 20% (0–40%), 20% (0–52%), and 30% (0–50%), respectively. All of the WPAI outcomes showed moderate to strong correlations with other generic and disease-specific measures (|r| = 0.43–0.75), except for absenteeism. Increasing disease activity and worse health status were significantly associated with increased impairments of work productivity and activity.

**Conclusion:**

This study highlights the negative effects of SAPHO syndrome on the work productivity and activity of patients, thus indicating good construct validity and discriminative ability of the WPAI. To reduce the economic burden, it is important to improve the work productivity and daily activity of patients by ameliorating clinical care.

**Supplementary information:**

The online version contains supplementary material available at 10.1186/s13023-022-02523-2.

## Key messages

1. Most employed SAPHO syndrome patients experienced decreased work productivity and activity.
2. There were statistically significant correlations between WPAI outcomes and other disease-related measures.
3. The WPAI questionnaire in SAPHO syndrome has good known-group validity.

**Supplementary information:**

The online version contains supplementary material available at 10.1186/s13023-022-02523-2.

## Introduction

Synovitis, acne, pustulosis, hyperostosis, and osteitis (SAPHO) syndrome is a rare disease characterized by osteoarticular and cutaneous manifestations. Osteitis and hyperostosis are regarded as the core pathophysiological changes of SAPHO syndrome [1], which may lead to bone pain and a loss of motor function. The symptoms of SAPHO syndrome usually appear in young and middle-aged adults [2] and persist during a relapsing-remitting disease course [3]. Therefore, patients with SAPHO syndrome may have an impairment of work productivity and activity.

To further understand this burden and to evaluate whether any intervention is effective for improving work productivity, a valid and reliable instrument is needed. The Work Productivity and Activity Impairment (WPAI) questionnaire is an instrument for measuring the impact of the disease on work productivity and activity, and it has been subsequently adapted for ankylosing spondylitis [4], rheumatoid arthritis [5], irritable bowel syndrome [6], and other chronic diseases [7].

The negative impacts of SAPHO syndrome on the quality of life of patients have been reported in Germany, and musculoskeletal symptoms are major factors influencing the overall disease burden [8]. However, no study has investigated the work productivity of patients with SAPHO syndrome. Therefore, the objective of this study was to provide an overview of work productivity and activity in patients with SAPHO syndrome and to investigate the relationship between the WPAI and other disease-related indicators.

## Methods

### Source of patients and patient-selection criteria

For this cross-sectional study, patients were recruited from Peking Union Medical College Hospital (PUMCH) (Beijing, China). After informed consent was obtained from each participant, they were asked to finish a questionnaire. Ethical approval was obtained from the Ethics Committee of PUMCH (Identifier: ZS-944).

#### Criteria for inclusion of patients

(1) a diagnosis of SAPHO syndrome according to the diagnosis criteria proposed by Kahn [9] in 2003; (2) symptoms such as bone pain persisting in the most recent month; and (3) age ≥ 9 years and age ≤ 75 years.

#### Criteria for exclusion of patients

(1) did not cooperate to participate in the questionnaire study (29 patients); and (2) completed an invalid questionnaire.

**Fig. 1:**
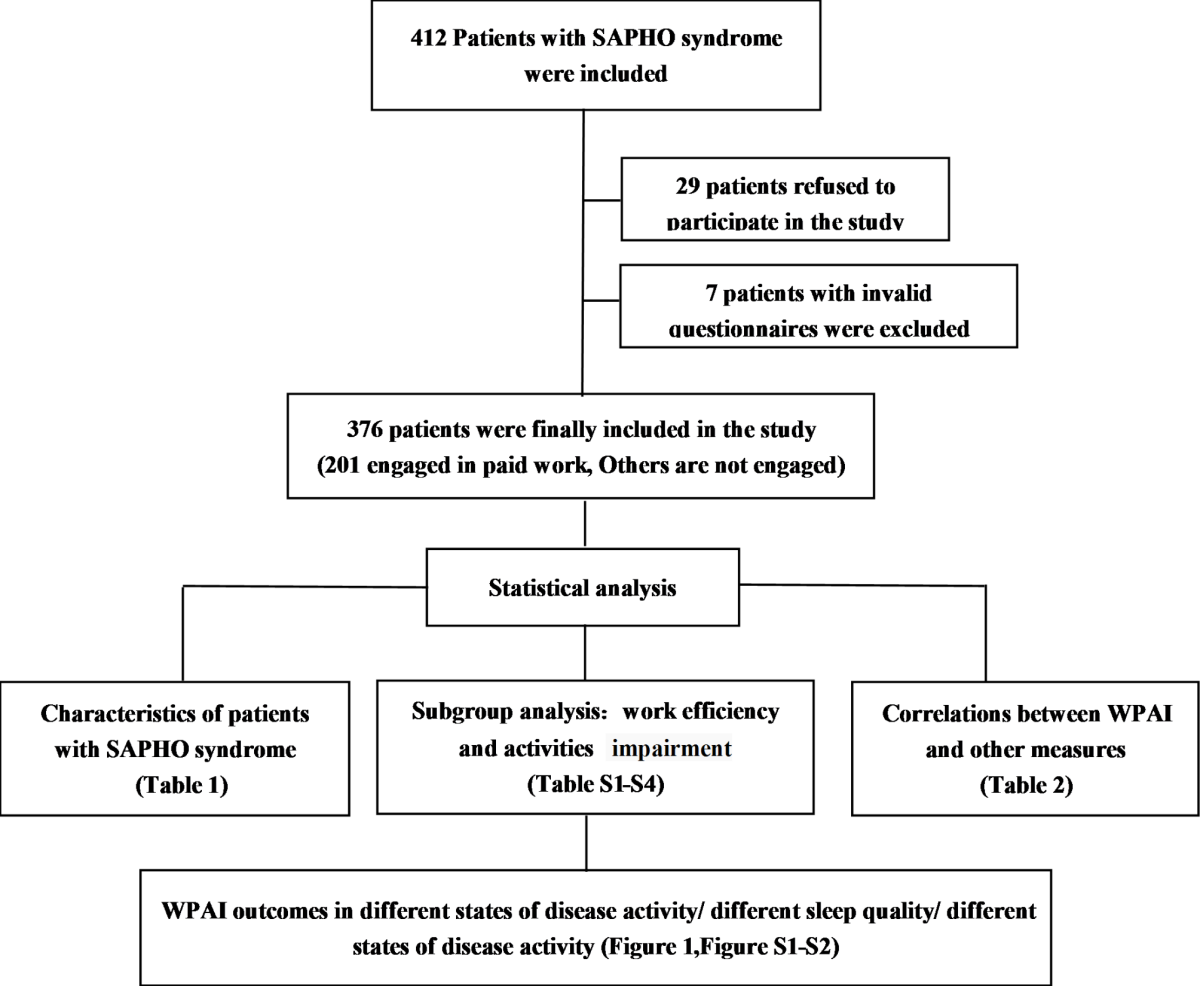
Flowchart of research methods and results

A total of 412 patients were initially recruited, and a total of 36 patients were excluded from the study. Finally, a total of 376 patients participated in the study. Figure 1 shows the overall framework of this study.

### Patient-reported outcome measures

The questionnaires to be completed by the patients consisted of demographic data, disease-specific measures, and general health measures. Demographic and clinical data included age, sex, marital status, height, weight, education, and employment. Disease activity and functional parameters were assessed by using the Bath Ankylosing Spondylitis Disease Activity Index (BASDAI) [10], the Bath Ankylosing Spondylitis Functional Index (BASFI) [11], and the Visual Analogue Scale (VAS) pain scores. Higher BASDAI and BASFI values indicate worse disease activity and worse functional impairment, respectively. BSADAI scores of 4 or greater suggest suboptimal control of the disease.

Health status was evaluated via the Short-Form 36 Health Survey (SF-36) [12], the Health Assessment Questionnaire for the Spondyloarthropathies (HAQ-S) [13], the Ankylosing Spondylitis Quality of Life Questionnaire (ASQOL) [14], and the EuroQol-5 Dimension Questionnaire (EQ-5D) [15]. The physical component scale (PCS) and the mental health component scale (MCS) were calculated from the SF-36. Higher scores on the SF-36 and EQ-5D represent higher functioning and better health, respectively, whereas higher scores on the HAQ-S and ASQOL reflect higher impairment and worse quality of life, respectively.

Depression, fatigue, sleep quality, and work productivity were quantified by using the Center for Epidemiological Studies Depression (CES-D) questionnaire [16], the Fatigue Severity Scale (FSS) [17], the Pittsburgh Sleep Quality Index (PSQI) [18], and the WPAI [19], respectively. Higher CES-D scores indicate worse depressive symptoms. Scores of FSS over 4 are considered to represent clinically meaningful fatigue. A global PSQI score of more than 5 defines a significant level of sleep disturbance, and higher scores indicate worse sleep quality.

The WPAI contains six questions assessing the impact of SAPHO syndrome on work productivity and daily activities during the last seven days, with questions including current employment status, working hours missed due to SAPHO syndrome, working hours missed for other reasons, the actual working hours, the degree of SAPHO syndrome that affected productivity while working, and the degree of SAPHO syndrome that affected regular activities. Four dimensions generated from these questions include absenteeism (percentage of working time that was missed due to a problem), presenteeism (percentage of impairment while working due to a problem), work productivity loss (percentage of overall work impairment due to a problem), and activity impairment (percentage of activity impairment due to a problem) [19]. These outcomes are expressed as percentages by multiplying the following scores by 100: (1) absenteeism = Q2/(Q2 + Q4); (2) presenteeism = Q5/10; (3) work productivity loss = Q2/(Q2 + Q4) + [(1-Q2/(Q2 + Q4)) * (Q5/10)]; and (4) activity impairment = Q6/10.

### Statistical analysis

Data are presented as the mean ± standard deviation (SD) for continuous variables with normality, as the median (interquartile range [IQR]) for abnormally distributed continuous variables, and as the number (percentage) for categorical variables. A Spearman’s rank correlation was used for the correlation analysis between the WPAI outcomes and other measures, including the BASDAI, BASFI, VAS pain score, HAQ-S, ASQOL, EQ-5D, SF-36 PCS, SF-36 MCS, PSQI, CES-D, and FSS. Correlations were considered as very strong (correlation coefficient r ≥ 0.8), strong (r = 0.6–0.79), moderate (r = 0.4–0.59), weak (r = 0.2–0.39), and negligible (r ≤ 0.19). Bonferroni corrections were made for the multiple comparisons. Wilcoxon rank-sum tests and nonparametric Kruskal‒Wallis tests were used for the comparison of the WPAI outcomes between groups that were divided by disease activity, fatigue, sleep quality, sex, marital status, body mass index (BMI), and age. Statistical analyses were performed via SPSS (version 25.0), and a two-sided P value of less than 0.05 was considered to be statistically significant.

## Results

### Patient characteristics

A total of 412 patients were initially recruited, 29 of whom rejected participation in the study. Seven patients were excluded because of the invalid completion of the questionnaire. The final sample that was used for the analyses included 376 participants, and the patient characteristics are shown in Table 1. Among the patients, 114 (30.3%) patients were male, and the mean age was 43.2 ± 12.3 years. The median disease duration of the participants was 43.0 (22.0–72.0) months. The most commonly affected area of osteitis and hyperostosis was the anterior chest wall (n = 354; 94.1%), followed by the axial skeleton, including the spine and sacroiliac joints (n = 233; 62.0%), and the peripheral bone and joint (n = 232; 61.7%). A total of 330 patients (87.8%) had cutaneous involvement, which was manifested as palmoplantar pustulosis (n = 294; 78.2%), psoriatic nail disease (n = 80; 21.3%), psoriasis vulgaris (n = 78; 20.7%), or severe acne (n = 50; 13.3%).

**Table 1:** Characteristics of patients with SAPHO syndrome

	All patients (n = 376)	Patients employed (n = 201)
Age, mean (SD), y	43.2 (12.3)	41.2 (8.6)
Male, n (%)	114 (30.3)	75 (37.3)
Married, n (%)	333 (88.6)	187 (93.0)
Smoking, n (%)	93 (24.7)	59 (29.4)
BMI, mean (SD)	23.7 (3.6)	23.8 (3.6)
VAS, n (%)		
> 5	71	26
≤5	305	175
ESR (mm/h)		
> 40	57	25
≤ 40	297	157
none	22	19
CRP (g/L)		
>10	112	56
≤10	242	131
none	22	14
Disease duration, median (IQR), m	43.0 (22.0–72.0)	39.5 (18.0–72.0)
Education years, median (IQR), y	12.0 (10.0–15.0)	14.0 (12.0–16.0)
Anterior chest wall lesions, n (%)	354 (94.1)	192 (95.5)
Axial skeleton involvement, n (%)	233 (62.0)	115 (57.2)
Peripheral bone and joint involvement, n (%)	232 (61.7)	124 (61.7)
Cutaneous involvement, n (%)	330 (87.8)	180 (89.6)
Palmoplantar pustulosis, n (%)	294 (78.2)	159 (79.1)
Severe acne, n (%)	50 (13.3)	31 (15.4)
Psoriasis vulgaris, n (%)	78 (20.7)	45 (22.4)
Psoriatic nail disease, n (%)	80 (21.3)	45 (22.4)
VAS pain score, median (IQR)	3 (1–5)	3 (1–4)
PSQI, median (IQR)	9 (6–11)	8 (5–10)
CES-D, median (IQR)	10.5 (3–20)	8 (3–18)
FSS, median (IQR)	4.2 (2.4–5.9)	4.0 (2.3–5.3)
BASDAI, median (IQR)	2.2 (1.0-3.7)	1.8 (0.9–3.4)
BASFI, median (IQR)	0.6 (0.0-1.8)	0.4 (0.0-1.1)
EQ-5D, mean (SD)	0.74 (0.13)	0.76 (0.09)
HAQ-S, median (IQR)	0.04 (0.00-0.24)	0.00 (0.00-0.14)
ASQOL, median (IQR)	3 (3–8)	2 (0–7)
SF-36 PCS, median (IQR), %	42.5 (32.0-50.8)	45.1 (36.1–52.6)
SF-36 MCS, median (IQR), %	51.0 (41.7–61.8)	53.2 (42.9–62.6)
Absenteeism, median (IQR), %	NA	0 (0–13)
Presenteeism, median (IQR), %	NA	20 (0–40)
Work productivity loss, median (IQR), %	NA	20 (0–52)
Activity impairment, median (IQR), %	30 (0–50)	20 (20–40)

BMI, Body Mass Index; VAS, Visual Analogue Scale; PSQI, Pittsburgh Sleep Quality Index; CES-D, Center for Epidemiological Studies Depression; FSS, Fatigue Severity Scale; BASDAI, Bath Ankylosing Spondylitis Disease Activity Index; BASFI, Bath Ankylosing Spondylitis Functional Index; EQ-5D, EuroQol-5 Dimension questionnaire; HAQ-S, Health Assessment Questionnaire for the Spondyloarthropathies; ASQOL, Ankylosing Spondylitis Quality of Life Questionnaire; SF-36, Short-Form 36 Health Survey; PCS, physical component summary; MCS, mental component summary; SD, standard deviation; IQR, interquartile range; CRP, C-reactive protein; ESR, erythrocyte sedimentation rate

## Work productivity and activity impairment

Patients who were still in paid employment at the time of recruitment accounted for 53.5% of the sample, and they demonstrated a median (IQR) of 0% (0–13%) absenteeism, 20% (0–40%) presenteeism, and 20% (0–52%) work productivity loss. The median (IQR) of activity impairment for all of the patients was 30% (0–50%). No differences in the WPAI were observed between the groups based on age, sex, or BMI (Supplementary Tables S1-S3). Patients who were not married had significantly higher absenteeism (mean rank: 127.96 vs. 98.98, P = 0.038) and work productivity loss (mean rank: 130.64 vs. 98.78, P = 0.046) than patients who were married (Supplementary Table S4), but no differences were observed for the remaining outcomes.

## Correlations between the WPAI and other measures

Correlations between the WPAI outcomes and other scores are shown in Table 2. Most disease-specific and general health measures demonstrated moderate to strong correlations with presenteeism (|r| = 0.48–0.67), work productivity loss (|r| = 0.43–0.61), and activity impairment (|r| = 0.50–0.75). There were weak correlations between absenteeism and other measures (|r| = 0.21–0.29). The correlations of the WPAI were positive with VAS, BASDIA, BASFI, PSQI, CES-D, FSS, HAQ-S, and ASQOL and negative with EQ-5D, SF-36 PCS, and MCS, thus indicating that an increased disease activity or worsened health status corresponded to increased work productivity and activity impairment. In addition, we examined the correlation between CRP/ESR values and WPAI scores in patients with a high CRP value. As shown in Table S5, we selected SAPHO syndrome patients (n = 25) with an ESR value greater than 40 mm/h who were engaged in work. We were surprised to find that ESR had a specific correlation with the work productivity loss score (r = 0.410, P = 0.042) and presenteeism score (r = 0.457, P = 0.022). Subsequently, we selected SAPHO syndrome patients (n = 56) with a CRP value greater than 10 g/L who were engaged in work. We also found that CRP had a correlation with the work productivity loss score (r = 0.320, P = 0.016), absenteeism score (r = 0.289, P = 0.031), and presenteeism score (r = 0.281, P = 0.036). Therefore, based on the above results, we inferred that CRP/ESR values are correlated with WPAI when patients have high CRP/ESR activity values.

**Table 2:** Spearman rank correlation coefficients between WPAI outcomes and other scores

	Absenteeism	Presenteeism	Work productivity loss	Activity impairment
Sample size	201	201	201	376
VAS pain score	0.24 ^*^	0.54 ^**^	0.49 ^**^	0.59^**^
PSQI	0.23 ^*^	0.57 ^**^	0.53 ^**^	0.58^**^
CES-D	0.25 ^*^	0.59 ^**^	0.55 ^**^	0.61^**^
FSS	0.27 ^*^	0.61 ^**^	0.56 ^**^	0.66^**^
BASDAI	0.26^*^	0.60 ^**^	0.55 ^**^	0.67^**^
BASFI	0.24 ^*^	0.58 ^**^	0.51 ^**^	0.68^**^
EQ-5D	-0.25 ^*^	-0.55 ^**^	-0.51 ^**^	-0.66 ^**^
HAQ-S	0.21 ^*^	0.48 ^**^	0.43 ^**^	0.64 ^**^
ASQOL	0.27 ^*^	0.63 ^**^	0.57 ^**^	0.69 ^**^
SF-36 PCS	-0.29 ^*^	-0.67 ^**^	-0.61 ^**^	-0.75 ^**^
SF-36 MCS	-0.29 ^*^	-0.48 ^**^	-0.49 ^**^	-0.50 ^**^

VAS, Visual Analogue Scale; PSQI, Pittsburgh Sleep Quality Index; CES-D, Center for Epidemiological Studies Depression; FSS, Fatigue Severity Scale; BASDAI, Bath Ankylosing Spondylitis Disease Activity Index; BASFI, Bath Ankylosing Spondylitis Functional Index; EQ-5D, EuroQol-5 Dimension questionnaire; HAQ-S, Health Assessment Questionnaire for the Spondyloarthropathies; ASQOL, Ankylosing Spondylitis Quality of Life Questionnaire; SF-36, Short-Form 36 Health Survey; PCS, physical component summary; MCS, mental component summary

* P < 0.01

** P < 0.001 (Bonferroni-adjusted α-level)

## Associations between the WPAI and known groups

**Fig. 2:**
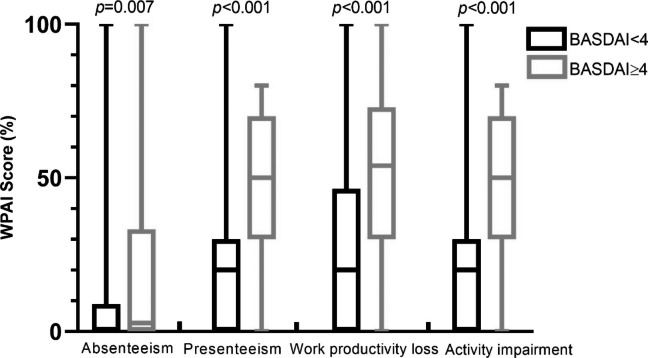
Box-plot of WPAI outcomes for patients with different states of disease activity as measured by BASDAI

Patients were divided into two groups according to disease activity, fatigue, and sleep quality. Figure 2 shows that each dimension of the WPAI outcomes was significantly higher among patients with active disease than among patients with lower disease activity (P values for the difference in absenteeism = 0.007 and P values for other WPAI outcomes < 0.001). Similar results were obtained in the comparisons of the WPAI outcomes according to the levels of fatigue (all P values < 0.001) and sleep quality (P values for the difference in absenteeism = 0.002 and P values for other WPAI outcomes < 0.001) (Supplementary Figures S1 and S2).

## Discussion

In this cross-sectional study, we investigated work productivity and activity impairment among patients with SAPHO syndrome by using a questionnaire survey. Our results showed that most employed patients experienced decreased productivity and activity, and there were statistically significant correlations between the WPAI outcomes and other disease-related measures, including disease activity, functional disability, quality of life, depression, fatigue, and sleep quality.

To our knowledge, this was the first study to investigate work productivity and activity impairment in a relatively large sample of patients with SAPHO syndrome. The morbidity of SAPHO syndrome is higher in women, and the ratio of males to females in this study was consistent with the epidemiology of SAPHO syndrome [2], which indicates that the sample has a good representation of the investigated population. When considering that a high risk for the occurrence of work productivity impairment in patients with SAPHO syndrome and a marked loss of work productivity may impose a substantial economic burden, it is important and valuable to improve the work productivity of SAPHO syndrome patients with aggressive treatments. Similar observations have been made in other spondyloarthropathies. For instance, Reilly et al. [4] reported that the median scores for absenteeism, presenteeism, overall work impairment, and activity impairment in patients with ankylosing spondylitis were 0%, 40%, 40%, and 60%, respectively, and patients with the more severe disease exhibited greater impairments in work productivity and daily activity than those patients with lesser disease severity (P < 0.001). The impairment of work productivity was significantly associated with disease activity, fatigue, and demographic factors in psoriatic arthritis [20–22]. The WPAI score of patients with SAPHO syndrome is different from that of patients with other autoimmune diseases. Ankylosing spondylitis (AS), psoriatic arthritis (PsA), and rheumatoid arthritis (RA) are all chronic inflammatory diseases. Long-term inflammation can cause movement disorders of the bones and joints, and peripheral joint erosion and sclerosis can lead to the loss of working ability [23–25]. However, SAPHO is a process of spontaneous remission and relapse. Most patients have a good prognosis and can maintain their working ability. Therefore, active treatment can alleviate disease activity during the active period of SAPHO syndrome, which helps in protecting working ability. In patients with psoriatic arthritis, work productivity was found to be significantly associated with sex and psoriasis area and severity index (PASI) [20]. Other studies have also found that work productivity impairment was significantly associated with disease activity, fatigue, and demographic factors in psoriatic arthritis [20–22]. However, in our study, we did not demonstrate that the sex and age of SAPHO syndrome patients were correlated with their WPAI scores. Nevertheless, we found that work productivity loss was significantly associated with marital status and health status. In addition, we similarly found that increased disease activity or worsened health status corresponded to increased work productivity and activity impairment. Our study also found significant differences in the WPAI scores between patients with different degrees of fatigue or sleep quality.

The appropriate construct validity of the WPAI in SAPHO syndrome was confirmed by moderate to strong correlations with all disease-specific and health status measures in terms of disease activity, functional disability, pain, quality of life, depression, fatigue, and sleep quality. However, weak correlations were observed between absenteeism and other measures. Similar results on the correlations between absenteeism and disease severity were observed in other studies [5, 26, 27]. Several interpretations can explain this apparent discrepancy. First, psychological factors such as pressure from career development, worries about unemployment, and economic stress can contribute to the difference in absenteeism and presenteeism. Second, it may be caused by different types of work (office vs. manual work) or different vacation times. Third, an imperfect social security system is expected to impact absenteeism more than presenteeism.

All four dimensions of the WPAI could discriminate between the two groups, as divided by disease activity, levels of fatigue, and sleep quality, which supports the known-group validity of the WPAI. Fatigue, sleep disturbance, psychological distress, and poor physical function are common problems among patients with SAPHO syndrome, and these symptoms can significantly influence the quality of life of patients and cause loss of work productivity. Similar results were observed in rheumatoid arthritis, psoriatic arthritis, spondyloarthropathies, systemic lupus erythematosus, and insomnia [28–30]. When regarding the association between autoimmune disorders and WPAI, the study of White D. demonstrated a significant reduction in the quality of life and work productivity in patients with ankylosing spondylitis [31]. When regarding the reliability, validity, and responsiveness of the work productivity and activity impairment questionnaires in ankylosing spondylitis, Reilly et al. found that patients with more severe AS (BASDAI > median) showed significantly greater impairments in work and daily activities than patients with less severe disease (P < 0.001) [32]. This conclusion is consistent with our findings. Similarly, we found that each dimension of the WPAI outcomes was significantly higher among patients with active disease than among patients with lower disease activity (P values for the difference in absenteeism = 0.007 and P values for other WPAI outcomes < 0.001). For rheumatoid arthritis (RA), drug therapy can alter the symptoms and WPAI scores. Among MTX-naive (SELECT-EARLY, MTX-naïve) and MTX-IR (SELECT-MONOTHERAPY, MTX-IR) patients with active RA, upadacitinib monotherapy at 15 or 30 mg for 12/14 weeks resulted in statistically significant and clinically meaningful improvements in WPAI compared with MTX alone [33].

Therefore, the impairment of work productivity and activity is not a single problem but a complex abnormal condition. In addition, we discovered that the impairments of work productivity and activity were independent of age, gender, and BMI. However, the absenteeism and work productivity loss in married patients were lower than in those patients who were not married, which may be attributable to the increasing financial stress or psychological support from the family.

## Limitations

This study also had some limitations. (1) In this study, we did not test the reliability of the WPAI among patients with SAPHO syndrome. However, the test-retest reliability of the WPAI has been well established in patients with different chronic diseases [34, 35]. (2) As this study was a cross-sectional study, the responsiveness assessed by measuring changes in the WPAI both before and after treatment was not accomplished. (3) Our study did not stratify disease according to time points (onset, during treatment, remission on the therapy, and remission off of the therapy). (4) The effect of comorbidities on patient work productivity was not analysed. Therefore, additional studies are needed to resolve these issues.

## Conclusion

This study highlights the negative effects of SAPHO syndrome on the work productivity and activity of patients, thus indicating good construct validity and discriminative ability of the WPAI. Increasing disease activity, poor quality of life, and worse health status are associated with higher impairments of work productivity and activity. To reduce the economic burden, it is important to improve the work productivity and daily activity of patients with active interventions.

Medians and interquartile ranges (%) for four dimensions of the WPAI in patients with different disease activities. The P values indicate a significant difference in scores between the two groups. Box-plot features represent the median (central line), upper and lower quartiles (box), and the maximum and minimum values of the data (bars).

## Electronic supplementary material

Below is the link to the electronic supplementary material.

Supplementary Material 1

## Data Availability

The datasets analyzed for this study are available from the corresponding author Dr. Chen Li (casio1981@163.com) upon reasonable request.
