# Supplementary material for: Work productivity and activity in patients with SAPHO syndrome: a cross-sectional observational study
Source: Orphanet J Rare Dis. 2022 Oct 21;17:381. doi: 10.1186/s13023-022-02523-2 (PMC9587537; doi:10.1186/s13023-022-02523-2)
Supplement: Supplementary file 1 — Supplementary Material 1 [file 13023_2022_2523_MOESM1_ESM.docx]

**Work Productivity and Activity Impairment in patients with SAPHO syndrome**

**Supplementary tables**

**Table S1. WPAI outcomes in different age groups**

| Age-group (y) | Absenteeism, median (IQR), % | Presenteeism, median (IQR), % | Work productivity loss, median (IQR), % | Activity impairment, median (IQR), % |
| --- | --- | --- | --- | --- |
| 20-24 | 2 (0-16) | 30 (10-50) | 30 (12-58) | 10 (0-40) |
| 25-29 | 6 (0-47) | 10 (0-30) | 25 (8-57) | 20 (0-30) |
| 30-34 | 0 (0-1) | 20 (10-50) | 20 (10-53) | 25 (0-50) |
| 35-39 | 0 (0-4) | 30 (10-50) | 30 (6-54) | 30 (10-50) |
| 40-44 | 0 (0-5) | 15 (0-40) | 16 (0-50) | 5 (0-48) |
| 45-49 | 0 (0-22) | 20 (0-50) | 21 (1-56) | 25 (0-48) |
| 50-54 | 0 (0-8) | 20 (0-30) | 20 (1-45) | 20 (0-35) |
| 55-59 | 0 (0-29) | 10 (0-40) | 13 (0-51) | 20 (0-40) |
| 60-64 | 0 (0-0) | 40 (0-80) | 40 (0-80) | 50 (0-100) |
| P value | 0.394 | 0.561 | 0.836 | 0.841 |

Abbreviations: IQR, interquartile range. P value derived from a non-parametric Kruskal-Wallis test.

**Table S2. WPAI outcomes in different gender groups**

|  | Male | Female | P |
| --- | --- | --- | --- |
| Absenteeism, median (IQR), % | 0 (0-13) | 0 (0-13) | 0.309 |
| Presenteeism, median (IQR), % | 20 (0-40) | 20 (0-50) | 0.696 |
| Work productivity loss, median (IQR), % | 20 (2-52) | 20 (0-52) | 0.848 |
| Activity impairment, median (IQR), % | 20 (0-40) | 30 (0-50) | 0.076 |

Abbreviations: IQR, interquartile range. P value derived from a Wilcoxon rank sum test.

**Table S3. WPAI outcomes in different BMI groups**

| BMI (kg/m^2^) | Absenteeism, median (IQR), % | Presenteeism, median (IQR), % | Work productivity loss, median (IQR), % | Activity impairment, median (IQR), % |
| --- | --- | --- | --- | --- |
| BMI<18.5 | 1 (0-41) | 15 (0-58) | 15 (0-80) | 40 (3-50) |
| BMI: 18.5-25 | 0 (0-12) | 20 (0-40) | 20 (2-51) | 20 (0-40) |
| BMI: 25-30 | 0 (0-10) | 20 (0-40) | 20 (0-53) | 20 (0-45) |
| BMI≥30 | 0 (0-36) | 25 (0-50) | 25 (1-74) | 45 (0-58) |
| P value | 0.777 | 0.954 | 0.951 | 0.417 |

Abbreviations: IQR, interquartile range; BMI, body mass index. P value derived from a non-parametric Kruskal-Wallis test. BMI<18.5, below normal weight; BMI ≥18.5 and <25, normal weight; BMI ≥25 and <30, overweight; BMI ≥30, obesity.

**Table S4. WPAI outcomes** **in different marital status groups**

|  | Patients married | Patients not married | P |
| --- | --- | --- | --- |
| Absenteeism, median (IQR), % | 0 (0-11) | 9 (0-50) | 0.038 |
| Presenteeism, median (IQR), % | 20 (0-40) | 30 (10-63) | 0.119 |
| Work productivity loss, median (IQR), % | 20 (0-50) | 55 (10-73) | 0.046 |
| Activity impairment, median (IQR), % | 20 (0-40) | 30 (7.5-50) | 0.293 |

Abbreviations: IQR, interquartile range. P value derived from a Wilcoxon rank sum test.

**Supplementary Figures**

**Figure S1**. Box-plot of WPAI outcomes for patients with different levels of fatigue as measured by FSS


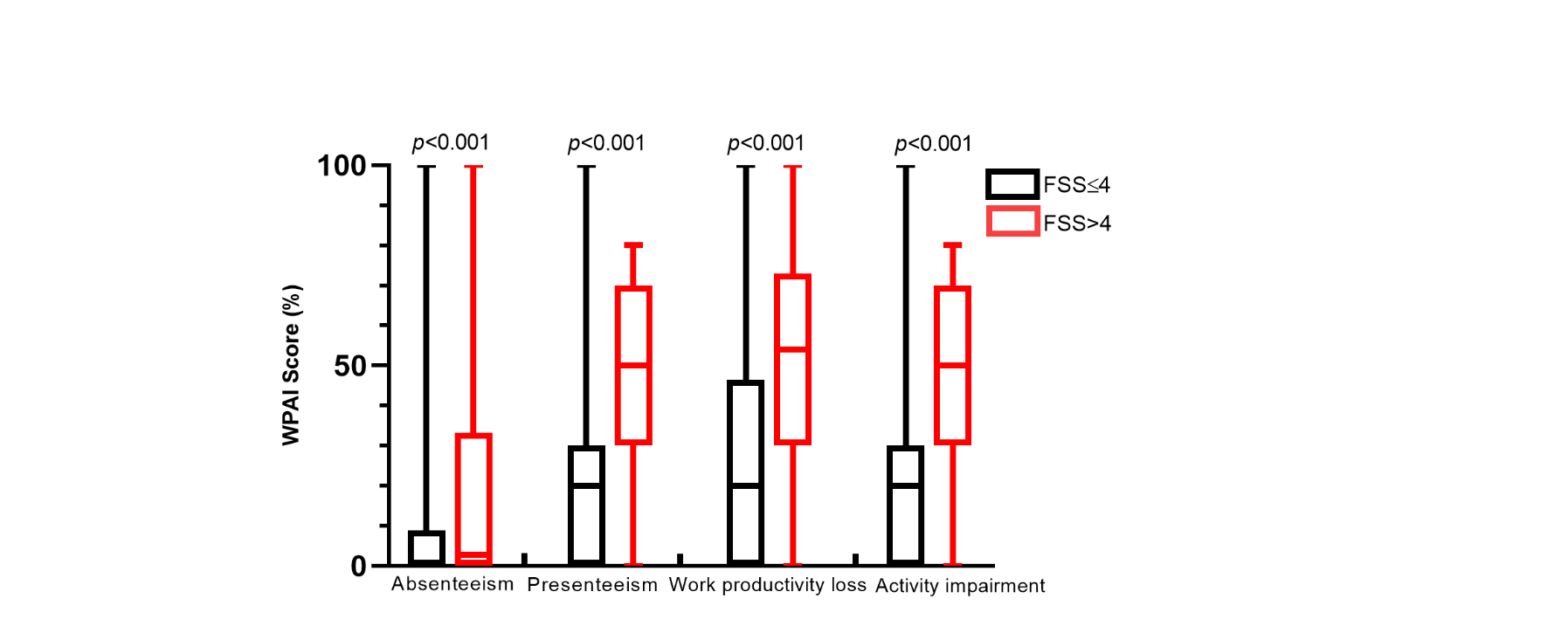


Median and interquartile range (%) for four dimensions of the WPAI in patients with different levels of fatigue. The p values indicate significant difference in scores between the two groups. Box-plot features represent the median (central line), upper and lower quartiles (box), and the maximum and minimum values of the data (bars).

**Figure S2**. Box-plot of WPAI outcomes for patients with different sleep quality measured by PSQI


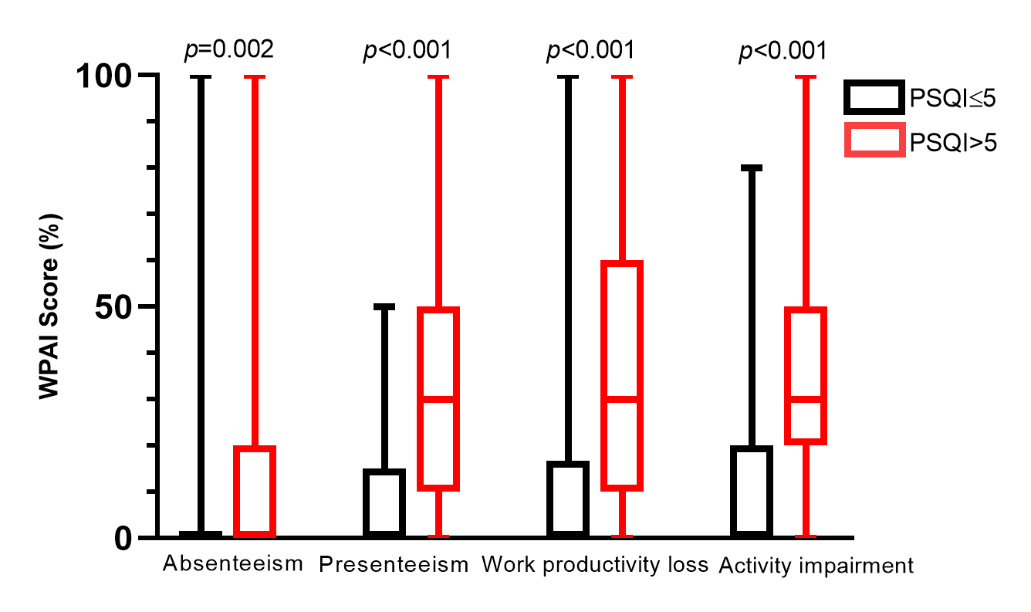


Median and interquartile range (%) for four dimensions of the WPAI in patients with different sleep quality. The p values indicate significant difference in scores between the two groups. Box-plot features represent the median (central line), upper and lower quartiles (box), and the maximum and minimum values of the data (bars).

**Table S5. Spearman rank correlation coefficients between WPAI outcomes and CRP/ESR**

|  | **Absenteeism** | **Presenteeism** | **work productivity loss** | **Activity impairment** |
| --- | --- | --- | --- | --- |
| ESR > 40 mm/h | 0.215 | 0.457 ^*^ | 0.410 ^*^ | 0.374 |
| CRP > 10 g/L | 0.289 ^*^ | 0.281 ^*^ | 0.320^*^ | 0.256 |

**Abbreviations:** Groups with ESR > 40 mm/h (n = 25); Groups with CRP > 10 g/L (n = 56);

* P<0.05.
